# In Vitro Anticancer Activity Screening of Novel Fused Thiophene Derivatives as VEGFR-2/AKT Dual Inhibitors and Apoptosis Inducers

**DOI:** 10.3390/ph15060700

**Published:** 2022-06-02

**Authors:** Rana M. Abdelnaby, Afaf A. El-Malah, Rasha R. FakhrEldeen, Marwa M. Saeed, Rania I. Nadeem, Nancy S. Younis, Hanaa M. Abdel-Rahman, Nehad M. El-Dydamony

**Affiliations:** 1 Pharmaceutical Chemistry Department, Faculty of Pharmacy, Heliopolis University, Cairo 11785, Egypt; 2 Pharmaceutical Organic Chemistry Department, Faculty of Pharmacy, King Abdulaziz University, Jeddah 21589, Saudi Arabia; afaf.elmalah@pharma.cu.edu.eg; 3 Pharmaceutical Organic Chemistry Department, Faculty of Pharmacy, Cairo University, Cairo 11562, Egypt; 4 Biochemistry Department, College of Pharmaceutical Sciences and Drug Manufacturing, Misr University for Science and Technology, 6th of October City 12585, Egypt; rasha.rashid@must.edu.eg; 5 Pharmacology and Toxicology Lecturer, Faculty of Pharmacy, Heliopolis University, Cairo 11785, Egypt; drmarwamsaeed@yahoo.com; 6 Pharmacology and Toxicology Department, Faculty of Pharmacy, Heliopolis University, Cairo 11785, Egypt; rania.ibrahim@hu.edu.eg; 7 Pharmaceutical Sciences Department, Faculty of Clinical Pharmacy, King Faisal University, Al-Ahsa, Al-Hofuf 31982, Saudi Arabia; nyounis@kfu.edu.sa; 8 Pharmacy Practice Department, Faculty of Pharmacy, Egyptian Russian University, Cairo 11829, Egypt; hanaa-ebido@eru.edu.eg; 9 Department of Forensic Medicine and Toxicology, Faculty of Medicine, Ain Shams University, Cairo 11562, Egypt; 10 Pharmaceutical Chemistry Department, College of Pharmaceutical Sciences and Drug Manufacturing, Misr University for Science and Technology, 6th of October City 12585, Egypt

**Keywords:** anticancer, apoptosis, thiophene, thienopyrimidine, VEGFR-2, AKT

## Abstract

Protein kinases are seen as promising targets in controlling cell proliferation and survival in treating cancer where fused thiophene synthon was utilized in many kinase inhibitors approved by the FDA. Accordingly, this work focused on adopting fused thienopyrrole and pyrrolothienopyrimidine scaffolds in preparing new inhibitors, which were evaluated as antiproliferative agents in the HepG2 and PC-3 cell lines. The compounds **3b** (IC_50_ = 3.105 and 2.15 μM) and **4c** (IC_50_ = 3.023 and 3.12 μM) were the most promising candidates on both cells with good selective toxicity-sparing normal cells. A further mechanistic evaluation revealed promising kinase inhibitory activity, where **4c** inhibited VEGFR-2 and AKT at IC_50_ = 0.075 and 4.60 μM, respectively, while **3b** showed IC_50_ = 0.126 and 6.96 μM, respectively. Moreover, they resulted in S phase cell cycle arrest with subsequent caspase-3-induced apoptosis. Lastly, docking studies evaluated the binding patterns of these active derivatives and demonstrated a similar fitting pattern to the reference ligands inside the active sites of both VEGFR-2 and AKT (allosteric pocket) crystal structures. To conclude, these thiophene derivatives represent promising antiproliferative leads inhibiting both VEGFR-2 and AKT and inducing apoptosis in liver cell carcinoma.

## 1. Introduction

One of the challenging areas in the drug development field is finding an effective treatment against cancer, a disease that represents the primary cause of death worldwide with millions of cases identified every year. Although many research efforts are focused on finding an effective treatment, there is still no 100% full cure. This may be due to the rapid changes occurring in the cancer microenvironment and the developing resistance against many anticancer therapies [1].

**Figure 1:**
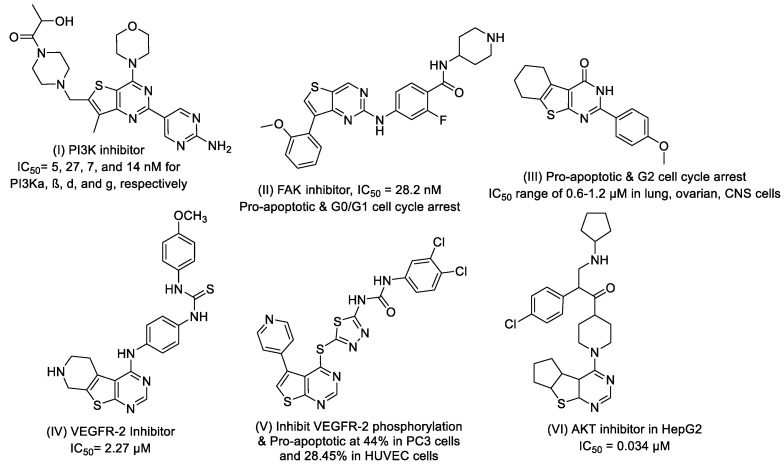
Thienopyrimidine derivatives with potential activities as anticancer agents; structures adopted from (**I**) [8,16], (**II**) [16,18], (**III**) [21], (**IV**) [8], (**V**) [23], and (**VI**) [25].

Fused thiophene derivatives, such as thienopyrimidines, have demonstrated efficacy as anticancer agents. They contain the pyrimidine moiety found in the natural nucleobase adenine, which occurs in DNA, RNA, ATP, and many bioactive molecules [2]. A large number of these derivatives have reached clinical trials, and many have been marketed [3,4]. This system has also attracted great attention due to its versatile synthetic approaches and the reported broad biological activities [5,6,7,8] that range from antihypertensive through alpha-1 adrenergic receptor antagonism [9], antiplatelet aggregation [10], antidepressant activity [11], treatment of erectile dysfunction, phosphodiesterase-5 inhibition [12], anti-inflammatory through COX-II inhibition [13], antimicrobial activity [14,15], and many other pledging activities [8], Noteworthy is the antiproliferative activity in which thienopyrimidine derivatives display potent activity with several mechanisms of action such as PI3K pathway inhibition [8,16,17], focal adhesion kinase (FAK) inhibition [18], kinases inhibitory activity against Tie-2 [19], cell cycle arrest and apoptosis induction [20,21,22]. Last but not least, they have promising anticancer activity by inhibiting the pathways under investigation,VEGFR-2 [23,24] and AKT-1 [25], that end in cancer cell proliferation, growth, and survival inhibition. Exemplified structures are presented in Figure 1.

The essential characteristics of a developing tumor involve sustaining proliferating signals, evading apoptosis, avoiding immune destruction, and inducing angiogenesis [26]. The vascular endothelial growth factor (VEGF) family was reported to be a core mediator in tumor progression and angiogenesis via interacting with VEGFR-1/2/3, the tyrosine kinase receptors, of which VEGFR-2 contributes mainly to increased vascular permeability, endothelial proliferation, invasion, and migration [27,28,29]. Thus, inhibiting the VEGF binding to its receptor either by neutralizing the ligand or the receptor by antibodies or direct inhibition of intracellular kinase domain through competing with the ATP-binding site is considered a very successful therapeutic strategy to halt tumor progress [27,29,30,31]. Additionally, VEGF binding to VEGFR-2 causes the activation of a central enzyme in cell survival, which is AKT (a serine/threonine kinase that belongs to the AGC kinase family) that is crucially involved in cell proliferation, evading apoptosis, protein synthesis, metabolism, angiogenesis, and migration [32,33,34,35,36,37]. AKT, this pivotal protein, is documented to be highly hyperactivated in various cancer types [34,38], which increases VEGF secretion and other growth factors creating a feedback loop that aids in developing cancer resistance to many antiangiogenic therapies [39,40].

Consequently, the dual inhibition of this axis VEGFR/AKT will trigger apoptosis at different focal points. This represents a successful tool in preventing tumorigenesis, proliferation, and survival with a better prognosis, particularly when taken as a part of a therapeutic process combination protocol [41].

**Figure 2:**
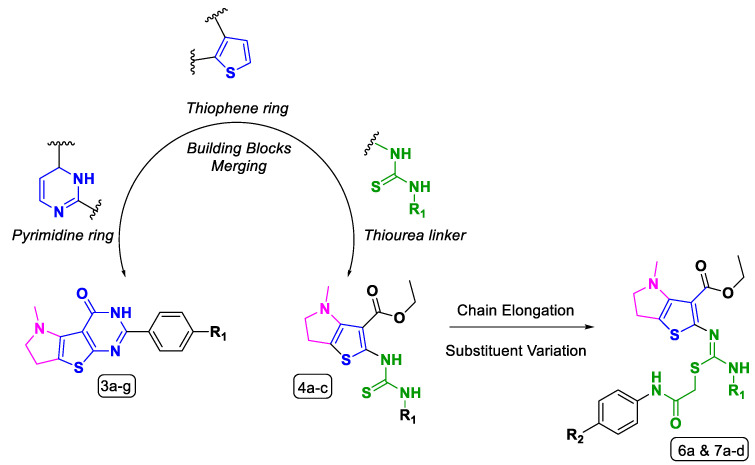
Development strategy of the novel thiophene candidates.

As illustrated in Figure 1, inhibitors acting on VEGFR-2 are characterized by a tripartite structure where the key features are a heteroaromatic moiety and an aromatic scaffold; both participate in hydrophobic interactions linked via three to five atoms in length incorporating a hydrogen bond acceptor/donor-forming group. Moreover, the AKT inhibitors share similar features to that of VEGFR-2 inhibitors, having a linear arrangement of pharmacophores [42]. The aforementioned data provided an enticing idea about designing a single molecule with dual activity on both proteins that represents a pivotal axis in cancer cell proliferation. This is done by merging these pharmacophores into one molecule by using the pyrrole–thiophene ring as the main nucleus fused with pyrimidine to give compounds **3a**–**g**. In addition, merging the pyrrole–thiophene ring with the thiourea linker gave **4a**–**c** analogs, and then the structure was extended by a terminal aromatic scaffold yielding **6a** and **7a**–**d** derivatives as depicted in Figure 2.

The novel compounds’ antiproliferative activity was investigated in HepG2 and PC-3 cell lines followed by a mechanistic evaluation for the nominated protein inhibition assay, cell cycle analysis, and apoptosis detection. Finally, molecular docking was conducted to assess the interaction possibilities with both VEFGR-2 and AKT-1.

## 2. Results and Discussion

### 2.1. Chemical Synthesis

**Scheme 1:**
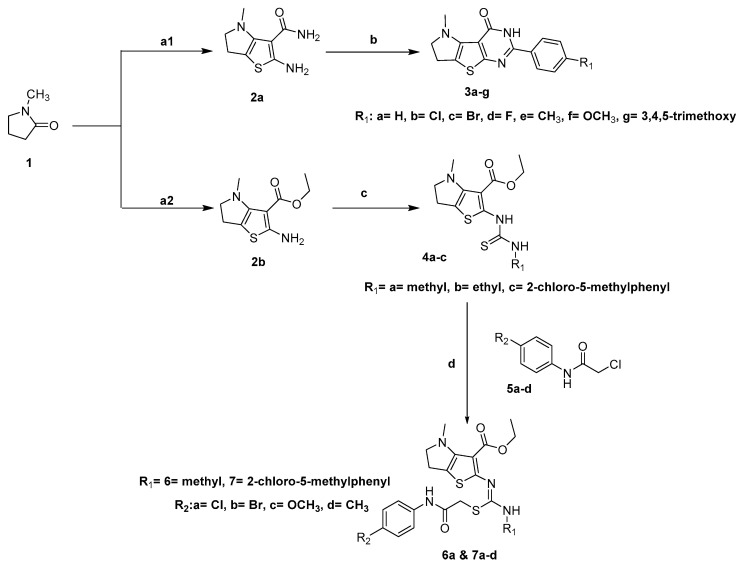
Conditions and reagents: (**a1**) cyanoacetamide, ethanol, morpholine, and sulfur; (**a2**) ethyl cyanoacetate, ethanol, morpholine, and sulfur; (**b**) aldehyde derivatives, DMF, C. HCl, and reflux for 24 h; (**c**) isothiocyanates, ethanol, and reflux for 8 h; (**d**) substituted aniline derivatives **5a**–**d**, potassium hydroxide, ethanol, and reflux for 18 h.

The approach to the synthesis of the entitled thiophenes was according to the reported procedures in the literature [3,43] and demonstrated in Scheme 1 where the spectral and physical analyses confirmed the proposed structures. Firstly, the starting compound *o*-amino amide derivative (**2a**) was reacted with different aromatic aldehydes yielding the fused pyrrolothienopyrimidine series (**3a**–**g**); their IR charts proved the absence of the primary amino forked peak, while ^1^H-NMR revealed the extra aromatic hydrogens at 7.23–7.99 ppm affected by the different parasubstitutions on the phenyl ring. For the open thiourea derivatives **6a** and **7a**–**d**, the *o*-amino ester (**2b**) was reacted with different aliphatic and aromatic isothiocyanates yielding the thieno [3,2-*b*]pyrrole-3-carboxylic acid ethyl ester series (**4a**–**c**) such as the IR, and the ^1^H-NMR showed the extra aliphatic and aromatic hydrogen substituted on the terminal amino group. The ^1^H-NMR showed the ethyl ester group at 1.04–1.31 ppm for the CH_3_- group, while the CH_2_-O group appeared at 4.00–4.50 ppm. Moreover, in compound **4a,** the aliphatic methyl attached to N of the thiourea terminal appeared at 2.51 ppm, while the 5-methyl group on the phenyl ring appeared at 2.19 ppm, and the aromatic hydrogen appeared at 7.21–7.37 ppm in compound **4c**. This was followed by a reaction with 2-chloro-*N*-(4-substituted-phenyl)-acetamide derivatives (**5a**–**d**) to alkylate the thiol group then the terminal amino group to attack the ester causing pyrimidine ring closure. However, it gave 2-[(4-substituted-phenyl carbamoyl)-methyl]-isothioureido analogs (**6a** and **7a**–**d**) without pyrimidine ring formation, and that was confirmed by IR and NMR analysis. This may be attributed to the bulkiness of the substituents on the terminal chain. The presence of the ethyl ester groups at 1.31–1.35 ppm for the terminal methyl (CH_3_-) and at 4.39–4.50 ppm for the -CH_2_-O group were revealed in ^1^H-NMR. In addition, the S-CH_2_ peak appeared at 4.3 ppm, and the aromatic hydrogens appeared at 7.11–8.01 ppm, which was confirmed by ^13^C-NMR analysis.

### 2.2. In Vitro Anticancer Activity Evaluation

#### 2.2.1. Antiproliferative Assay in HepG2 and PC-3 Cells

The antiproliferative activity of the thiophene compounds **3a**–**g**, **4a**–**c**, **6a**, and **7a**–**d** was evaluated by an in vitro MTT colorimetric assay on two oncogenic cell lines: liver (HepG2) and prostate (PC-3). The cytotoxic effect was calculated as the inhibitory concentration (IC_50_) in µM required for inhibiting the growth of 50% of the oncogenic cells taking doxorubicin as the reference compound. The results presented in Table 1 show that most of the novel thiophene derivatives possess moderate to high cytotoxic activity whether on the liver or prostate or both cell lines.

By looking at the thienopyrimidine series (**3a**–**g**), more derivatives showed higher activity against HepG2 cells than prostate cancer PC-3 cells. The best compound in this series was the chloro derivative **3b** with IC_50_ of 3.105 ± 0.14 μM and 2.15 ± 0.12 μM on HepG2 and PC-3, respectively. By comparing the derivatives in this series to the unsubstituted analog **3a**, it was found that substituting the phenyl ring with electron-withdrawing groups enhanced the activity on PC-3 cells, while on HepG2 cells, the inhibitory activity was better by substituting the phenyl ring at the para position with chloro, methoxy, and trimethoxy groups. The trimethoxy analog **3g** showed high inhibitory activity on HepG2 with IC_50_ of 3.77 ± 0.17 μM and moderate inhibition on PC-3 cells, while the methoxy derivative **3f** with IC_50_ of 4.296 ± 0.2 μM and 7.472 ± 0.42 μM resulted in high cytotoxicity on HepG2 and PC-3, respectively. Moreover, the bromo substitution in **3c** resulted in less cytotoxicity on PC-3 and a loss of activity against HepG2 cells, while the fluoro derivative **3d** showed moderate activity against both cell lines. The substitution with an electron-donating group such as the methyl group in analog **3e** resulted in lowering the cytotoxic activity on both cell lines.

For the open thieno[3,2-*b*]pyrrole series **4a**–**c**, the 3-methyl derivative **4a** showed high inhibitory activity on PC-3 cells, although it has no activity against HepG2, while the 3-ethyl derivative **4b** was less active in the two cell lines. The aromatic substitution in 2-chloro-5-methyl phenyl derivative **4c** resulted in marked activity on both cell lines.

In the 2-isothiouredo-4-methyl-thieno[3,2-*b*]pyrrole series **6a** and **7a**–**d**, the substitution of **4a** with *N*-(4-chloro-phenyl)-acetamide led to a marked increase in the cytotoxic activity of **6a**. While substitution of **4c** with different *N*-(4-substituted-phenyl)-acetamide resulted in decreasing the inhibitory activity except for **7d,** which has the electron-donating methyl group on the side chain that gave good inhibitory activity compared to doxorubicin.

The aforementioned results illustrate that compounds **3b** and **4c** are the most promising cytotoxic agents on both cell lines and will be subjected to a thorough mechanistic study to understand their anticancer activity. However, before doing this, their selective toxicity and, thus, their safety were evaluated by measuring the IC_50_ values against the normal nononcogenic cell line (Wl38) by an MTT assay as listed in Table 1. The SI calculated and reported by [44] was higher than two, which indicated that compounds **3b** and **4c** possess good safety. Compound **3b** gave SI of 6, and compound **4c** gave SI = 12 showing better selectivity toward cancer cells than doxorubicin with SI = 5.

#### 2.2.2. Assessment of VEGFR-2/AKT Axis Inhibition

##### VEGFR-2 Inhibition Assay

**Figure 3:**
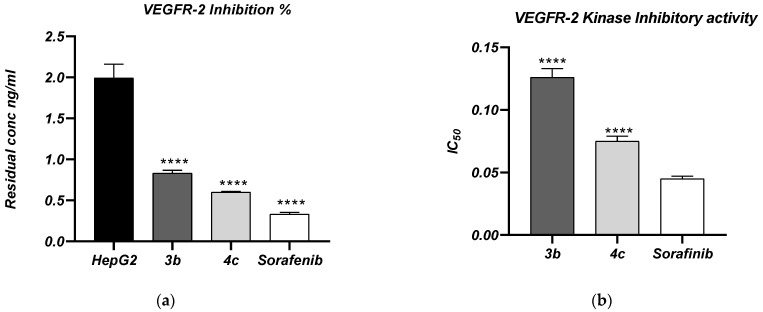
(**a**) Effect of treatment of HepG2 cell line with **3b** and **4c** compounds on VEGFR2 residual concentration using ELISA. (**b**) Kinase inhibitory activity assay (IC_50_ in μM) of **3b** and **4c** on VEGFR-2 using ELISA and taking sorafenib as the reference compound. Values are given as mean ± S.D. for groups of 3. (****) significantly different from HepG2 group at *p* < 0.0001.

The inhibitory effect of the two selected candidates **3b** and **4c** on VEGFR-2 was evaluated in HepG2 cells at their formerly determined IC_50_. Compound **3b** showed moderate inhibition by 58.3%, while **4c** resulted in a significant inhibitory effect by 70% relative to the reference compound sorafenib, which resulted in 83.3% phosphorylation inhibition as compared to HepG2 cells. Furthermore, the assessment of their kinase inhibitory activity revealed a very promising in vitro effect with IC_50_ values of 0.126 μM for compound **3b** and 0.075 μM for **4c** that represent potent inhibition approaching sorafenib with 0.045 μM as shown in Figure 3 and higher potency than compound **IV** (IC_50_ values of 2.27 μM) illustrated in Figure 1.

##### AKT-1 Inhibition Assay

**Figure 4:**
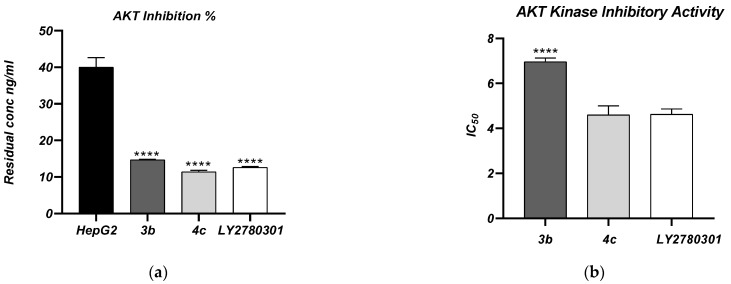
(**a**) Effect of treatment of HepG2 cell line with **3b** and **4c** compounds on AKT-1 residual concentration using ELISA. (**b**) Kinase inhibitory activity assay (IC_50_ in μM) of **3b** and **4c** on AKT using ELISA and taking LY2780301 as the reference compound. Values are given as mean ± S.D. for groups of 3. (****) significantly different from HepG2 group at *p* < 0.0001.

To investigate the success of the design strategy developed in this work, the effect of the promising candidates **3b** and **4c** was assessed on AKT-1, a crucial protein, by determining their phosphorylation inhibition rate in HepG2 cells and measuring their biochemical kinase inhibitory activity. As illustrated in Figure 4, compound **3b** caused a 63% inhibition rate, while **4c** was more promising with a 71.6% inhibition rate that was better than LY2780301 with 68.6%. Moreover, AKT-1 kinase activity was inhibited at IC_50_ of 6.96 μM by compound **3b** and IC_50_ = 4.60 μM of **4c** revealing a similar inhibition to the reference drug LY2780301 giving IC_50_ of 4.62 μM. The data obtained from the protein inhibition assays demonstrated that both compounds have the required features of VEGFR-2 and AKT-1 and are very promising dual-acting inhibitors on VEGFR-2 and AKT-1 proteins, especially **4c** with similar effects to the reference compound.

#### 2.2.3. The Effect of **3b** and **4c** on Cell Cycle Phases and Their Apoptosis Induction

##### Cell Cycle Analysis

**Figure 5:**
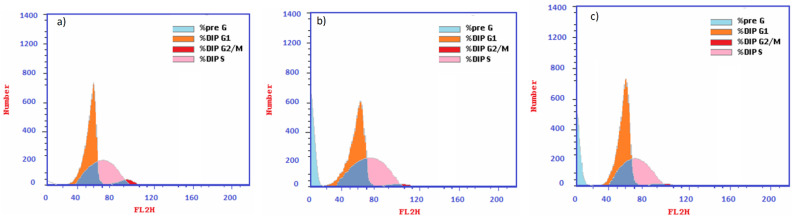
Cell cycle analysis and DNA content in different cell cycle phases after treating HepG2 cells with **3b** and **4c** for 48 h. (**a**) Control HepG2 cells; (**b**) compound **3b**; (**c**) compound **4c**.

AKT-1 is known for its downstream activation of several proteins involved in cell proliferation and apoptosis evasion. In addition, it is reported to be involved in G1–S checkpoint transition and proliferation [45]. Thus, the AKT-1 inhibition and antiproliferative effect of **3b** and **4c** on the cell cycle progress was assayed by flow cytometry at their IC_50_ values shown in Table 2 and Figure 5.

For compound **3b**, there was a notable buildup of cells in the S phase with 56.19%, while a low concentration of cells transitioned to the G2/M phases with 4.39% indicating cell cycle arrest at the S phase. For compound **4c**, there was a higher accumulation of cells in G0/G1 of 53.28% and a very low concentration in the G2/M phase indicating arrest at the G1/S phase.

##### Apoptosis Detection

**Figure 6:**
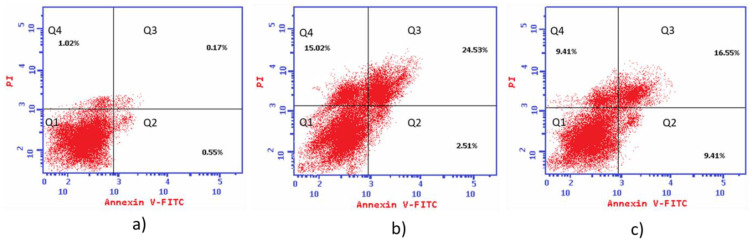
The proapoptotic effect of the novel compounds **3b** and **4c** on HepG2 cells after 48 h against control, untreated cells. Q1: viable cells; Q2: early apoptotic; Q3: late apoptotic; and Q4: necrotic cell content. (**a**) Control HepG2 cells; (**b**) compound **3b**; (**c**) compound **4c.**

The proapoptotic effect of **3b** and **4c** was evaluated after causing cell cycle arrest by treating HepG2 cells for 48 h with their IC_50_ values. Annexin V/PI staining with flow cytometric analysis was adapted to measure early and late apoptotic cells and necrotic cells as presented in Table 3 and Figure 6.

Treating HepG2 cells with **3b** for 48 h resulted in an enhanced apoptotic effect with an elevated proportion in early and late apoptotic cells exhibiting 5-fold and 144-fold, respectively. While compound **4c** causes a higher proportion in early apoptotic cells with 7-fold over untreated cells and 97-fold in late apoptotic cells. For necrotic cells, **4c** had less of a necrotic effect than **3b,** which was consistent with their selectivity against normal cells (WI38) in Table 1 signaling a better safety profile for **4c** over **3b**.

These data are consistent with previously reported effects of thiophene derivatives to be effective as anticancer through cell cycle arrest and apoptosis induction [21,22,23].

#### 2.2.4. Caspase-3 Assay

**Figure 7:**
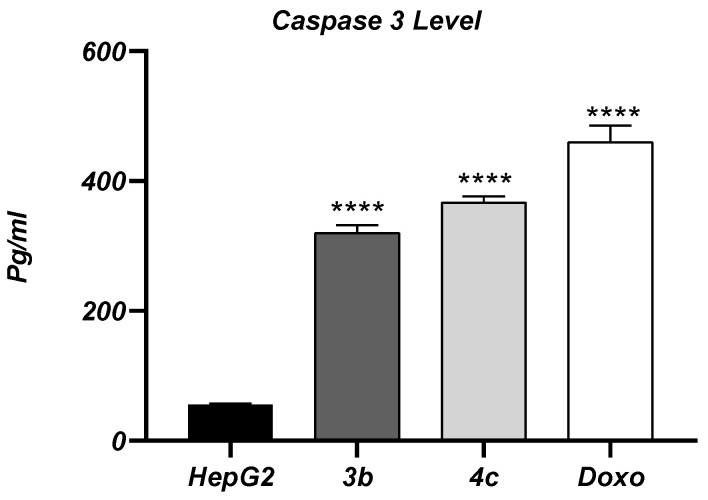
The effect of treating HepG2 cells with **3b** and **4c** compounds on caspase-3 concentration using ELISA. Values are given as mean + S.D. for groups of 3. (****) significantly different from HepG2 group at *p* < 0.0001.

Apoptosis is known to be carried out through three different pathways: intrinsic, extrinsic, and granzyme pathways. One of the controlling proteins in apoptosis is the cysteine protease named caspase with the chief executer caspase 3 [46,47]. Accordingly, assessing the effect of **3b** and **4c** on the active caspase-3 level was done in HepG2 cells taking doxorubicin as the reference drug. The results shown in Figure 7 demonstrate a five-fold increase in its level with **3b** treatment and a six-fold increase after **4c** treatment relative to doxorubicin that showed a seven-fold over the untreated cells.

### 2.3. Molecular Modeling

**Figure 8:**
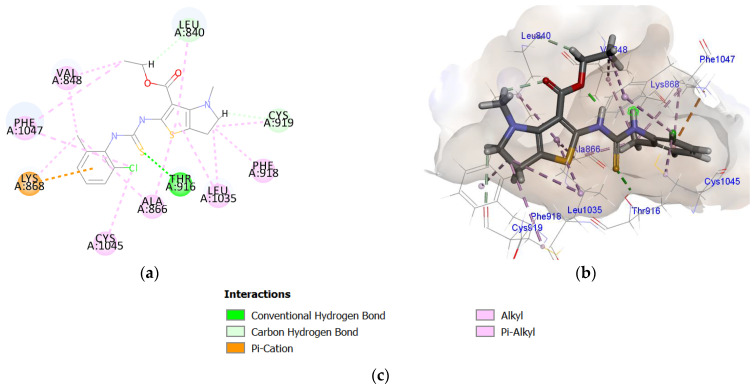
Binding interaction of **4c** inside VEGFR-2 active site with CDOCKER score of −11.20 Kcal/mol. (**a**) The 2D interaction diagram; (**b**) the 3D interaction diagram; and (**c**) the binding interactions codes.

**Figure 9:**
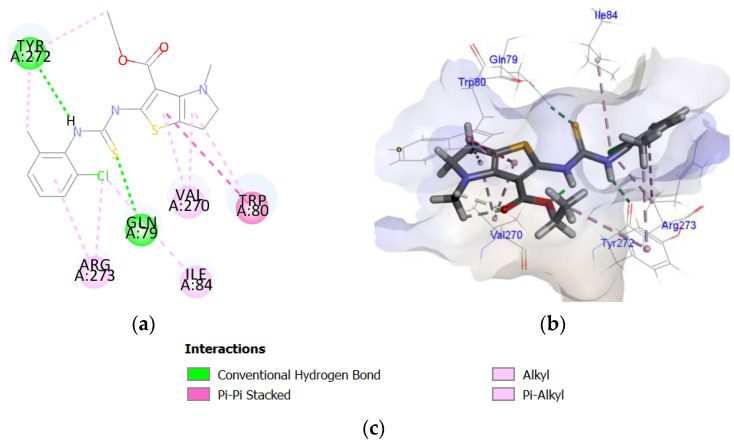
Binding interaction of **4c** inside AKT-1 active site with CDOCKER score of −10.16 Kcal/mol. (**a**) the 2D interaction diagram; (**b**) the 3D interaction diagram; and (**c**) the binding interactions codes.

Performing docking simulations inside the nominated protein binding sites clarified the molecular interactions of the novel derivatives as depicted in Figure 8 and Figure 9.

#### 2.3.1. VEGFR-2 Docking

Molecular docking studies of thienopyrrole ethyl ester **4c** derivative against the tyrosine kinase receptor VEGFR-2 (PDB code: 3EWH) [48] showed a hydrogen bond (2.20 Å) between the S of the thiourea linker and the Thr916 residue in addition to two carbon–hydrogen bonds between the H of ethyl ester with Leu840 and the H of pyrrole with Cys919. The phenyl ring imparted a pi–cation interaction with Lys868. The thiophene ring and chloride atom incorporated pi–alkyl interactions with Ala866 and Cys1045 residues. Moreover, there were two intramolecular interactions where one of the thiourea NH engaged in an H-bond with the oxygen of the ester group; the other is the pi–alkyl interaction between 5-methyl on the phenyl ring with the oxygen atom of the carbonyl ester. The binding revealed a preferred pattern with the crucial amino acids in the receptor active pocket where the thienopyrrole nucleus was embedded in the front hydrophobic pocket, and the substituted phenyl was rooted in the back pocket.

#### 2.3.2. AKT Docking

The AKT structure consists of three conserved domains: (a) N-terminal Pleckstrin Homology (PH domain); (b) kinase domain (KD domain), and (c) C-terminal hydrophobic motif. AKT inhibitors are either ATP-competitive or allosteric inhibitors where they work at the interface of the PH and KD domains. Docking simulations of compound **4c** in the AKT allosteric active site (PDB code: 4EJN) [42] was performed to study its inhibitory mechanism. The results illustrate a binding mode with the key amino acids in which two hydrogen bonds were formed between the S atom of the thiourea linker with Gln79 (2.58 Å) and the hydrogen of the NH to the carbonyl oxygen of the Tyr272 (2.56 Å). The thienopyrrole nucleus was pi–pi stacked through its thiophene ring to Trp80 in the PH domain, on the other hand, the substituted phenyl shared an alkyl interaction with Arg273 and Ile84 in the c-terminal motif.

#### 2.3.3. ADME Calculation for Compound **4c**

To predict the druggability of the best compound **4c**, the molecular properties were measured and were consistent with the Lipinski criteria that states: “for a drug candidate to be orally bioavailable it should have a molecular mass fewer than 500 Daltons, H-bond donors (HBD) ≤ 5, H-bond acceptors (HBA) ≤ 10, and log *p* < 5. [49]” Our compounds with Log *p* = 5.75 may show good absorption levels and penetration into cancer cells. In addition, **4c** has two H-bond donors, four H-bond acceptors, and seven rotatable bonds.

## 3. Material and Methods

### 3.1. Chemistry

The spectral analysis for structure confirmations were carried out in Cairo University, Egypt. The FTIR spectra were determined in Shimadzu IR 435 spectrophotometer using potassium bromide discs in v_max,_ cm^−1^. A Bruker spectrometer (Bruker Corp., Billerica, MA, USA) recorded ^1^H-NMR (DMSO at 400 MHz) and ^13^C-NMR (DMSO at 100 MHz) spectra, while a Hewlett Packard 5988 spectrometer was used to detect the mass spectra.

The starting compounds (**2a** and **2b**) were prepared according to the reported thiophene synthesis adopting the Gewald procedures [9,43], and the linker derivatives 2-Chloro-N-(4-substituted-phenyl)-acetamide **5a**–**d** were as reported by [50].

**2-Amino-4-methyl-5,6-dihydro-4*H*-thieno[3,2-b]pyrrole-3-carboxamide (2a):** Yellowish white powder, mp. = 121–123 °C, yield % = 42.8; IR: 3400, 3390 (2 NH_2_), 2951 (CH_aliphatic_), 1666 (C=O); ^1^H-NMR (δ and ppm) 2.51 (s, 3H, and N-CH_3_), 3.42–3.47 (t, 2H, and CH_2_-CH_2_-N), 3.75–3.77 (t, 2H, and CH_2_-CH_2_-N), 7.00, 7.302 (s, 4H, 2 NH_2_, and D_2_O exchangeable); MS (*m/z*): 197 (M+); Elemental analysis of C_8_H_11_N_3_OS (197): C, 48.71; H, 5.62; and N, 21.30 found: C, 48.95; H, 5.75; and N, 21.19.

**Ethyl 2-amino-4-methyl-5,6-dihydro-4H-thieno[3,2-b]pyrrole-3-carboxylate (2b):** Buff powder, mp. = 130 °C, yield % = 50; IR: 3352 (NH_2_), 2981–2870 (CH_aliphatic_), 1720 (C=O); ^1^H-NMR (400 MHz and CDCl_3_-d_6_) δ 1.45 (t, 3H, and CH_3_-CH_2_-O), 2.45 (s, 3H, and N-CH_3_), 2.98–3.02 (t, 2H, and CH_2_-CH_2_-N), 3.68–3.72 (t, 2H, and CH_2_-CH_2_-N), 4.23–4.27 (q, 2H, and CH_3_-CH_2_-O), 7.00 (s, 2H, NH, and D_2_O exchangeable); MS (*m/z*): 226 (M+), 227 (M+1); Elemental analysis of C_10_H_14_N_2_O_2_S (226): C, 53.08; H, 6.24; and N, 12.38 found: C, 53.31; H, 6.38; and N, 12.62.

**Preparation procedure of the 2-(substituted)-5-methyl-3,5,6,7-tetrahydro-4*H*-pyrrolo[2′,3′:4,5]thieno[2,3-*d*]pyrimidin-4-one derivatives (3a–g):** The appropriate aldehyde derivatives (0.03 mmol) were added to the amino amide derivative **2a** (0.01 mmol, 1.97 g) dissolved in 10 mL DMF with 0.2 mL C.HCl. The reaction was refluxed for 24 h and cooled, and the precipitate was crystallized from acetone.

**5-Methyl-2-phenyl-3,5,6,7-tetrahydro-4H-pyrrolo[2′,3′:4,5]thieno[2,3-d]pyrimidin-4-one (3a):** Yellowish brown powder, mp. > 250 °C, yield % = 83; IR: 3352 (NH), 2981–2870 (CH_aliphatic_), 1720 (C=O); ^1^H-NMR (δ, ppm) 2.51 (s, 3H, and CH_3_-N), 2.94 (t, 2H, and CH_2_-CH_2_-N), 3.70 (t, 2H, and CH_2_-CH_2_-N), 7.23–7.29 (m, 2H, C3, and C5-H_Ar_), 7.36–7.40 (t, 1H, and C4-H_Ar_) and 7.60–7.62 (d, 2H, C2, and C6-H_Ar_) and 7.99 (s, 1H, NH, and D_2_O exchangeable); ^13^C-NMR (δ and ppm) 24.79 (CH_2_-CH_2_-N and pyrrolidine), 43.18 (CH_3_-N and pyrrolidine), 44.22 (CH_2_-CH_2_-N and pyrrolidine), 126.10–143.69 (Ar-C), 169.86 and 174.41 (2C and pyrimidine); MS (*m/z*): 283 (M+), 284 (M+1); Elemental analysis of C_15_H_13_N_3_OS (283): C, 63.58; H, 4.62; and N, 14.83; found: C, 63.42; H, 4.89; and N, 15.04.

**2-(4-Chlorophenyl)-5-methyl-3,5,6,7-tetrahydro-4H-pyrrolo[2′,3′:4,5]thieno[2,3-d]pyrimidin-4-one (3b):** Yellowish powder, mp. = 168 °C, yield % = 72; IR: 3394 (NH), 3024 (CH_Ar_), 2966 (CH_aliphatic_), 1624 (C=O), 1519–1446 (C=C, Ar); ^1^H-NMR (δ, ppm) 2.55 (s, 3H, and CH_3_-N), 3.12–3.15 (t, 2H, and CH_2_-CH_2_-N), 3.48–3.50 (t, 2H, and CH_2_-CH_2_-N), 7.27–7.49 (m, 4H, and H_Ar_), and 8.12 (s, 1H, NH, and D_2_O exchangeable); MS (*m/z*): 317 (M+), 319 (M+2); Elemental analysis of C_15_H_12_ClN_3_OS (317): C, 56.69; H, 3.81; and N, 13.22; found: C, 56.78; H, 3.94; and N, 13.40.

**2-(4-Bromophenyl)-5-methyl-3,5,6,7-tetrahydro-4H-pyrrolo[2′,3′:4,5]thieno[2,3-d]pyrimidin-4-one (3c):** Yellowish orange powder, mp. = 113–114 °C, yield % = 74; IR: 3456 (NH), 3020 (CH_Ar_), 2875 (CH_aliphatic_), 1668 (C=O) 1583–1485 (C=C and Ar); ^1^H-NMR (δ and ppm) 2.51 (s, 3H, and CH_3_-N), 2.90–3.00 (t, 2H, and CH_2_-CH_2_-N), 3.78–3.82 (t, 2H, and CH_2_-CH_2_-N), 7.77–7.87 (m, 4H, and H_Ar_), and 7.99 (s, 1H, NH, and D_2_O exchangeable); MS (*m/z*): 360 (M+), 362 (M+2); Elemental analysis of C_15_H_12_BrN_3_O_S_ (362): C, 49.74; H, 3.34; and N, 11.60 found: C, 49.95; H, 3.61; and N, 11.87.

**2-(4-Fluorophenyl)-5-methyl-3,5,6,7-tetrahydro-4H-pyrrolo[2′,3′:4,5]thieno[2,3-d]pyrimidin-4-one (3d):** Yellowish white powder, mp. = 108–110 °C, yield % = 90; IR: 3352 (NH), 3050 (CH_Ar_), 2981–2870 (CH_aliphatic_), 1720 (C=O); ^1^H-NMR (δ, ppm) 2.53 (s, 3H, and CH_3_-N), 2.90–2.93 (t, 2H, and CH_2_-CH_2_-N), 3.65 (t, 2H, and CH_2_-CH_2_-N), 7.83–7.86 (m, 4H, and H_Ar_), and 7.91 (s, 1H, NH, and D_2_O exchangeable); MS (*m/z*): 301 (M+), 302 (M+1); Elemental analysis of C_15_H_12_FN_3_OS (301): C, 59.79; H, 4.01; and N, 13.94; found: C, 59.87; H, 4.19; and N, 14.16.

**5-Methyl-2-(p-tolyl)-3,5,6,7-tetrahydro-4H-pyrrolo[2′,3′:4,5]thieno[2,3-d]pyrimidin-4-one (3e):** Buff powder, mp. = 155–156 °C, yield % = 75.5; IR: 3441 (NH), 3032 (CH_Ar_), 2970 (CH_aliphatic_), 1666 (C=O); ^1^H-NMR (δ and ppm) 2.39 (s, 3H, and 4-CH_3_-Ph), 2.45 (s, 3H, and CH_3_-N), 2.90–3.00 (t, 2H, and CH_2_-CH_2_-N), 3.18 (t, 2H, and CH_2_-CH_2_-N), 7.05 (d, 2H, C3, and C5-H_Ar_), 7.97–7.99 (d, 2H, C2, and C6-H_Ar_), and 7.5 (s, 1H, NH, and D_2_O exchangeable); MS (*m/z*): 297 (M+); Elemental analysis of C_16_H_15_N_3_O_2_S (297): C, 61.32; H, 4.82; and N, 13.41; found: C, 61.17; H, 4.98; and N, 13.65.

**2-(4-Methoxyphenyl)-5-methyl-3,5,6,7-tetrahydro-4H-pyrrolo[2′,3′:4,5]thieno[2,3-d]pyrimidin-4-one (3f):** Buff powder, mp. = 117–118 °C, yield % = 63; IR: 3421 (NH), 3035 (CH_Ar_), 2997 (CH_aliphatic_), 1604 (C=O), 1523–1450 (C=C and Ar); ^1^H-NMR (δ and ppm) 2.51 (s, 3H, and CH_3_-N), 2.95 (t, 2H, and CH_2_-CH_2_-N), 3.48 (t, 2H, and CH_2_-CH_2_-N), 3.78 (s, 3H, and O-CH_3_), 6.91–6.93 (d, 2H, C3, and C5-H_Ar_), 7.27–7.29 (d, 2H, C4, and C6-H_Ar_), 7.5 (s, 1H, NH, and D_2_O exchangeable); ^13^C-NMR (δ and ppm) 43.59 (CH_2_-CH_2_-Nand pyrrolidine), 44.43 (2C, CH_3_-N, CH_2_-CH_2_-N, and pyrrolidine), 55.70 (4-Ph-OCH_3_), 113.66–136.04 (Ar-C), 159.81 and 186.76 (2C and pyrimidine); MS (*m/z*): 313 (M+), 314 (M+1); Elemental analysis of C_16_H_15_N_3_O_2_S (313): C, 61.32; H, 4.82; and N, 13.41; found: C, 61.17; H, 4.98; and N, 13.65.

**5-Methyl-2-(3,4,5-trimethoxyphenyl)-3,5,6,7-tetrahydro-4H-pyrrolo[2′,3′:4,5]thieno[2,3-d]pyrimidin-4-one (3g):** Brownish yellow powder, mp. = 114–115 °C, yield % = 60; IR: 3441 (NH), 2966 (CH_aliphatic_), 1624 (C=O) cm^−1^; ^1^H-NMR (δ, ppm) 2.55 (s, 3H, and CH_3_-N), 2.73–2.77 (t, 2H, and CH_2_-CH_2_-N), 3.36–3.48 (t, 2H, and CH_2_-CH_2_-N), 3.68, 3.77, and 3.80 (3s, 9H, and Ph-OCH_3_), 6.58 (s, 2H, C2, and C6 Ar-H), 6.83 (s, 1H, NH, and D_2_O exchangeable); ^13^C-NMR (δ and ppm) 43.22 (CH_2_-CH_2_-N and pyrrolidine), 44.22 (2C, CH_3_-N, CH_2_-CH_2_-N, and pyrrolidine), 56.18 (2C, 3- and 5-Ph-OCH_3_), 60.81 (4-Ph-OCH_3_), 103.15–138.81 (Ar-C), 153.05 and 188.78 (2C and pyrimidine); MS (*m/z*): 373 (M+), 374 (M+1); Elemental analysis of C_18_H_19_N_3_O_4_S (373): C, 57.90; H, 5.13; and N, 11.25; found: C, 58.05; H, 5.26; and N, 11.43.

**The preparation procedure of 2-(3-substituted-thioureido)-4-methyl-5,6-dihydro-4H-thieno[3,2-b]pyrrole-3-carboxylic acid ethyl ester (4a–c):** The amino ester derivative **2b** (0.01 mmol, 2.26 g) with the appropriate isothiocyanates (0.01 mmol) and KOH were refluxed for 8 h in absolute ethanol. The precipitate product was crystallized from ethanol.

**Ethyl 4-methyl-2-(3-methylthioureido)-5,6-dihydro-4H-thieno[3,2-b]pyrrole-3-carboxylate (4a):** Buff powder, mp. = 132–134 °C, yield % = 81.5; IR: 3417 and 3290 (2NH), 2978–2881 (CH_aliphatic_), 1635 (C=O) cm^−1^; ^1^H-NMR (δ and ppm) 1.04–1.08 (t, 3H, and CH_3_-CH_2_-O-C=O), 2.51 (s, 6H, and 2 CH_3_-N), 3.33–3.41 (t, 2H, and CH_2_-CH_2_-N), 3.43–3.45 (t, 2H, and CH_2_-CH_2_-N), 4.35 (q, 2H, and CH_3_-CH_2_-O-C=O), 8.70 and 9.85 (s, 2H, 2NH, and D_2_O exchangeable); MS (*m/z*): 298 (M+), 299 (M+1); Elemental analysis of C_12_H_17_N_3_O_2_S_2_ (299): C, 48.14; H, 5.72; and N, 14.03; found: C, 48.58; H, 5.60; and N, 14.21.

**Ethyl 2-(3-ethylthioureido)-4-methyl-5,6-dihydro-4H-thieno[3,2-b]pyrrole-3-carboxylate (4b):** Buff powder, mp. = 135–136 °C, yield % = 80.1; IR: 3417 and 3320 (2NH), 2981–2827 (CH_aliphatic_), 1693 (C=O), 2353 and 1384 (-SH); ^1^H-NMR (δ and ppm) 1.20 (m, 6H, CH_3_-CH_2_-N, and CH_3_-CH_2_-O), 2.51 (s, 3H, and N-CH_3_), 3.22–3.34 (m, 4H, and CH_2_-CH_2_-N), 3.40–4.00 (m, 4H, CH_3_-CH_2_-N, and CH_3_-CH_2_-O), 8.50 and 9.60 (s, 2H, 2NH, and D_2_O exchangeable); MS (*m/z*): 313 (M+); Elemental analysis of C_13_H_19_N_3_O_2_S_2_ (313): C, 49.82; H, 6.11; and N, 13.41; found: C, 48.58; H, 5.60; and N, 14.21.

**Ethyl 2-(3-(2-chloro-5-methylphenyl)thioureido)-4-methyl-5,6-dihydro-4H-thieno[3,2-b]pyrrole-3-carboxylate (4c):** Yellowish powder, mp. = 94 °C, yield % = 82.5; IR: 3340 and 3182 (2NH), 3001 (CH_Ar_), 2978 (CH_aliphatic_), 1693 (C=O), 1527–1411 (C=C and Ar); ^1^H-NMR (δ and ppm) 1.31–1.35 (t, 3H, and CH_3_-CH_2_-O-C=O), 2.19 (s, 3H, and 5-Ph-CH_3_), 2.20 (s, 3H, and CH_3_-N), 3.33–3.52 (m, 4H, and CH_2_-CH_2_-N), 4.39–4.50 (q, 2H, and CH_3_-CH_2_-O-C=O), 7.21–7.37 (m, 3H, and H_Ar_), 10.58 and 10.71 (s, 2H, 2NH, and D_2_O exchangeable); ^13^C-NMR (δ and ppm) 14.17 (CH_3_-CH_2_-O-C=O), 18.28 (5-Ph-CH_3_), 18.49 (CH_2_-CH_2_-N), 66.62 (CH_3_-N and pyrrolidine), 67.26 (CH_2_-CH_2_-N), 68.49 (CH_3_-CH_2_-O-C=O), 124.51–141.59 (Ar-C), 155.04 (O=C-O ester), 190.14 (S=C-NH); MS (*m/z*): 409 (M+), 411 (M+2); Elemental analysis of C_18_H_20_ClN_3_O_2_S_2_ (410): C, 52.74; H, 4.92; and N, 10.25; found: C, 52.95; H, 5.06; and N, 10.51.

**The preparation procedure of 2-{3-substitutted-2-[(4-susbstituted-phenylcarbamoyl)-methyl]-isothioureido}-4-methyl-5,6-dihydro-4H-thieno[3,2-b]pyrrole-3-carboxylic acid ethyl ester derivatives (6a and 7a–d):** Compounds **4a** and **4c** (0.01 mmol) with potassium hydroxide (0.02 mmol, 1.12 g) were suspended in absolute ethanol and stirred for 2 h. The substrates **5a**–**d** (0.012 mmol) were added, and reflux was continued for 18 h. The solid produced was crystallized from *n*.hexane.

**Ethyl (Z)-2-((((2-((4-chlorophenyl)amino)-2-oxoethyl)thio)(methylamino)methylene)amino)-4-methyl-5,6-dihydro-4H-thieno[3,2-b]pyrrole-3-carboxylate (6a):** Yellowish powder, mp. = 140–141 °C, yield % = 75; IR: 3332 and 3242 (2NH), 3118–3059 (CH_Ar_), 2983–2939 (CH_aliphatic_), 1695 and 1658 (2C=O), 1533–1446 (C=C and Ar); ^1^H-NMR (δ and ppm) 1.30–1.33 (t, 3H, and CH_3_-CH_2_-O-C=O), 3.33 (s, 6H, and 2 CH_3_-N), 3.50–3.56 (t, 4H, and CH_2_-CH_2_-N), 4.00 (s, 2H, and S-CH_2_-C=O), 4.28–4.34 (q, 2H, and CH_3_-CH_2_-O-C=O), 7.34–7.62 (m, 4H, and H_Ar_), 10.68 and 10.92 (2H, 2NH, and D_2_O exchangeable); ^13^C-NMR (δ, ppm) 14.28 (CH_3_-CH_2_-O-C=O), 18.28 (CH_2_-CH_2_-N), 18.88 (2C, S-CH_2,_ and CH_3_-NH), 59.12 (2C, CH_3_-N, CH_2_-CH_2_-N, and pyrrolidine), 62.98 (CH_3_-CH_2_-O-C=O), 121.22–137.76 (Ar-C), 156.03 (O=C-O ester), 160.87 (N-C=N), 187.93 (NH-C=O, amide); MS (*m/z*): 467 (M+), 469 (M+2); Elemental analysis of C_20_H_23_ClN_4_O_3_S_2_ (467): C, 51.44; H, 4.96; and N, 12.00; found: C, 51.52; H, 4.71; and N, 12.27.

**Ethyl (Z)-2-((((2-chloro-5-methylphenyl)amino)((2-((4-chlorophenyl)amino)-2-oxoethyl)thio)methylene)amino)-4-methyl-5,6-dihydro-4H-thieno[3,2-b]pyrrole-3-carboxylate (7a):** Yellowish powder, mp. = 82–83 °C, yield % = 72.5; IR: 3332 and 3182 (2NH), 3000 (CH and Ar), 2981–2870 (CH_aliphatic_), 1701 and 1670 (2C=O), 1527–1404 (C=C and Ar); ^1^H-NMR (δ and ppm) 1.31–1.35 (t, 3H, and CH_3_-CH_2_-O-C=O), 2.19 (s, 3H, and 5-Ph-CH_3_), 2.20 (s, 3H, CH_3_-N, and pyrrolidine), 2.22–2.25 (t, 2H, and CH_2_-CH_2_-N), 4.29 (t, 2H, and CH_2_-CH_2_-N), 4.30–4.34 (s, 2H, and S-CH_2_-C=O), 4.39–4.50 (q, 2H, and CH_3_-CH_2_-O-C=O), 7.23–8.01 (m, 7H, and H_Ar_), 10.58 and 10.71 (s, 2H, 2NH, and D_2_O exchangeable); ^13^C-NMR (δ and ppm) 14.28 (CH_3_-CH_2_-O-C=O), 18.42 (5-Ph-CH_3_), 18.48 (2C, CH_2_-CH_2_-N, and S-CH_2_), 62.96 (CH_3_-N and pyrrolidine), 66.56 (CH_2_-CH_2_-N), 66.97 (CH_3_-CH_2_-O-C=O), 122.33–156.01 (Ar-C), 160.90 (O=C-O and ester), 188.77 (N-C=N), 190.33 (NH-C=O and amide); MS (*m/z*): 578 (M+); Elemental analysis of C_26_H_26_Cl_2_N_4_O_3_S_2_ (578): C, 54.07; H, 5.72l; and N, 9.70; found: C, 54.25; H, 5.88; and N, 9.89.

**Ethyl (Z)-2-((((2-((4-bromophenyl)amino)-2-oxoethyl)thio)((2-chloro-5-methylphenyl)amino)methylene)amino)-4-methyl-5,6-dihydro-4H-thieno[3,2-b]pyrrole-3-carboxylate (7b):** Reddish brown powder, mp. >250 °C, yield % = 79; IR: 3350 and 3292 (2NH), 3010 (CH_Ar_), 2980 (CH_aliphatic_), 1699 (C=O) 1529–1485 (C=C and Ar); ^1^H-NMR (δ and ppm) 1.31–1.35 (t, 3H, and CH_3_-CH_2_-O-C=O), 2.19 (s, 3H, and 5-Ph-CH_3_), 2.20 (s, 3H, CH_3_-N, and pyrrolidine), 2.68–2.73 (t, 2H, and CH_2_-CH_2_-N), 4.30–4.32 (s, 2H, and S-CH_2_-C=O), 4.41–4.50 (m, 4H, CH_3_-CH_2_-O-C=O, and CH_2_-CH_2_-N), 7.25–7.77 (m, 7H, and Ar-H), 10.44 and 10.66 (s, 2H, 2NH, and D_2_O exchangeable); ^13^C-NMR (δ and ppm) 14.51 (CH_3_-CH_2_-O-C=O), 18.42 (5-Ph-CH_3_), 18.48 (2C, CH_2_-CH_2_-N, and S-CH_2_), 62.96 (CH_3_-N and pyrrolidine), 66.56 (CH_2_-CH_2_-N), 66.97 (O=C-O-CH_2_-CH_3_), 122.68–130.20 (Ar-C), 156.34 (O=C-O ester), 175.78 (N-C=N), 190.33 (NH-C=O and amide); MS (*m/z*): 623 (M+), 625 (M+2), 627 (M+4); Elemental analysis of C_26_H_26_BrClN_4_O_3_S_2_ (622): C, 50.21; H, 4.21; and N: 9.01; found: C, 49.94; H, 4.53; and N, 9.28.

**Ethyl (Z)-2-((((2-chloro-5-methylphenyl)amino)((2-((4-methoxyphenyl)amino)-2-oxoethyl)thio)methylene)amino)-4-methyl-5,6-dihydro-4H-thieno[3,2-b]pyrrole-3-carboxylate (7c):** Yellowish powder, mp. = 110–112 °C, yield% = 71; IR: 3340, 3228 (2NH), 3062 (CH_Ar_), 2974–2904 (CH_aliphatic_), 1793 and 1635 (2C=O), 1589–1454 (C=C and Ar) cm^−1^; ^1^H-NMR (δ and ppm) 1.31–1.35 (t, 3H, and CH_3_-CH_2_-O-C=O), 2.19 (s, 3H, and 5-Ph-CH_3_), 2.20 (s, 3H, CH_3_-N, and pyrrolidine), 2.23–2.27 (t, 2H, and CH_2_-CH_2_-N), 4.26–4.33 (m, 5H, CH_2_-CH_2_-N, and 4-Ph-OCH_3_), 4.39–4.50 (m, 4H, CH_3_-CH_2_-O-C=O, and S-CH_2_-C=O), 7.11–7.85 (m, 7H, and Ar-H), 10.44 and 10.58 (s, 2H, NH, and D_2_O exchangeable); ^13^C-NMR (δ and ppm) 14.5 (CH_3_-CH_2_-O), 18.42 (5-Ph-CH_3_), 20.91 and 21.20 (2C, S-CH_2_, and CH_2_-CH_2_-N), 62.96 (2C, CH_3_-N, and 4-Ph-OCH_3_), 66.56 (CH_2_-CH_2_-N), 66.98 (O=C-O-CH_2_-CH_3_), 119.81–139.20 (Ar-C), 186.67 (O=C-O ester), 188.78 (N=C-S-CH_2_), 190.34 (NH-C=O, amide); MS (*m/z*): 573 (M+), 574 (M+1); Elemental analysis of C_27_H_29_ClN_4_O_4_S_2_ (573): C, 56.58; H, 5.10; and N, 9.78; found: C, 56.83; H, 5.23; and N, 9.89.

**Ethyl (Z)-2-((((2-chloro-5-methylphenyl)amino)((2-oxo-2-(p-tolylamino)ethyl)thio)methylene)amino)-4-methyl-5,6-dihydro-4H-thieno[3,2-b]pyrrole-3-carboxylate (7d):** Yellowish powder, mp. = 118–120 °C, yield % = 79.5; IR: 3417 and 3236 (2NH), 3032 (CH_Ar_), 2958–2858 (CH_aliphatic_), 1678 and 1643 (2C=O), 1589–1454 (C=C and Ar); ^1^H-NMR (δ and ppm) 1.31–1.35 (t, 3H, and CH_3_-CH_2_-O-C=O), 2.19 (s, 3H, and 5-Ph-CH_3_), 2.20 (s, 3H, CH_3_-N, and pyrrolidine), 2.25–2.33 (m, 5H, 4′-Ph-CH_3_, and CH_2_-CH_2_-N), 4.25–4.29 (t, 2H, and CH_2_-CH_2_-N), 4.39 (s, 2H, and S-CH_2_-C=O), 4.41–4.50 (m, 2H, and CH_3_-CH_2_-O-C=O), 7.13–7.85 (m, 7H, and H_Ar_), 10.44 and 10.58 (s, 2H, 2NH, and D_2_O exchangeable); ^13^C-NMR (δ and ppm) 14.63 (CH_3_-CH_2_-O), 18.40 (2C, 4-Ph-CH_3,_ and 5-Ph-CH_3_), 21.98 and 21.20 (S-CH_2_ and CH_2_-CH_2_-N), 66.58 (2C, CH_3_-N, pyrrolidine, and CH_2_-CH_2_-N), 66.99 (O=C-O-CH_2_-CH_3_), 120.55–139.20 (Ar-C), 159.91 (S-C=N), 186.80 (O=C-O ester), 190.34 (NH-C=O, amide); MS (*m/z*): 557 (M+), 559 (M+2); Elemental analysis of C_27_H_29_ClN_4_O_3_S_2_ (557): C, 58.21; H, 5.25; and N: 10.06; found: C, 58.45; H, 5.42; and N, 10.28.

### 3.2. Biological Activity Investigation

#### 3.2.1. MTT Antiproliferative Assay

All cell lines (HepG2, PC-3, and nontumorigenic WI38) were supplied from VACSERA (Cairo, Egypt), cultured in DMEM (Invitrogen/Life Technologies, Waltham, MA, USA) at a density of 1.2–1.8 × 10,000 cells/well in a volume of 100 µL of the growth medium, and then incubated for 24 h, and an MTT assay was done as reported by [51,52]. Cell viability is presented as the control and the drug concentrations that cause 50% of the inhibition of cell proliferation (IC_50_).

#### 3.2.2. Inhibition Assays for VEGFR-2 and AKT-1 Proteins

##### Cell-Based Evaluation of Inhibition Percentage in HepG2 Cells

VEGFR-2 phosphorylation was assayed using VEGFR2 Antibody (Phospho-Tyr951) (OAEC00085, AVIVA SYSTEM BIOLOGY) [53], while Akt-1 was assayed by Mouse/Human/Rat Phospho-AKT1 (Ser473) (Cell-Based Phosphorylation ELISA) ELISA Kit-LS-F1447 (LSBio) according to manufacturer’s instructions [54].

##### Evaluation of the Inhibitory Effect on VEGFR-2 and AKT Kinase Activity

The nominated protein kinase activity inhibition by the novel compounds was determined quantitively by the VEGFR2 (KDR) Kinase Assay Kit (Catalog # 40325, BPS, Bioscience, San Diego, CA, USA) against VEGFR-2 [55,56] and Akt Kinase Activity Assay Kit (ab139436, ABCAM, USA) for AKT-1 enzyme on cell lysate according to the reported procedures [57,58]. The outcomes were expressed as IC_50_ values (mean standard deviation) calculated from the dose–response curves and their linear regression equations. Sorafenib was used as the reference in the VEGFR-2 assay, and LY2780301 was used in the AKT assay.

#### 3.2.3. Flow Cytometric Cell Cycle Analysis and Apoptosis Induction

Analyzing the DNA content in the cell cycle phases by flow cytometry was conducted using FACSCalibur (Becton Dickinson, Franklin Lakes, NJ, USA) following the manufacturer’s manual. The cell cycle analysis procedures were as reported by [59,60] on HepG2 cells after their treatment with the most promising derivatives for 48 h. Further assessment of the proapoptotic effect was determined by the Annexin V-FITC/PI apoptosis detection kit (Catalog # K101-25, BD Biosciences, San Diego, CA, USA) [61,62].

#### 3.2.4. Caspase-3 Evaluation

The colorimetric ELISA assay for active caspase-3 detection was carried out utilizing Invitrogen™ Caspase-3 (Cleaved) Human ELISA-Kit (KHO1091, CA, USA) according to the manufacturer’s procedures [63]. ROBONIK P2000 ELISA reader was used at 450 nm, and the standard curve was obtained by plotting the mean absorbance of each standard concentration against human caspase-3 concentrations.

### 3.3. Molecular Modeling

Molecular modeling was done according to the reported procedures of [42,51,64] using DiscoveryStudio 4.1 software (Accelrys, Inc., San Diego, CA, USA). The X-ray 3D structures of VEGFR-2 (PDB ID: 3EWH) [48] and AKT-1 (PDB ID: 4EJN) [42,65] were obtained from the PDB site (http://www.rscb.org/pdb (accessed on 30 April 2022) and prepared for docking by cleaning the protein and fixing missing chains. The CHARMm forcefield was applied, and energy was minimized. The binding pockets were defined, and the validation step was done following the reported steps. Then, the docking of the prepared ligands into the 3D structures of the proteins was carried out assuming flexible ligand-rigid receptor docking using CDOCKER protocol. The best 10 poses were studied, and the one with the best score and orientation was chosen.

### 3.4. Statistical Analysis

Analysis was conducted using GraphPad Prism v5, where *p* values ≤ 0.05 were regarded as statistically significant using one-way ANOVA followed by Tukey–Kramer as the post hoc test.

## 4. Conclusions

By synthesizing novel thiophene derivatives and evaluating their anticancer activities, promising agents were discovered as antiproliferative in two cell lines, namely HepG2 and PC-3, which are prevalent in a high percentage worldwide. An MTT assay revealed that most of the compounds are effective against both cell lines, especially the thienopyrimidine analog **3b** and thiouriedo **4c,** which were further proven to be excellent kinase inhibitors against VEGFR-2 and AKT-1 proteins that represent a pivotal axis in modulating cell proliferation. Inhibiting this axis in HepG2 cells resulted in S-phase arrest and apoptosis induction via caspase-3 activation. Molecular docking showed binding interactions with key amino acids inside both active sites.

These findings encourage further studies on the most active compound **4c** as an adjuvant anticancer with VEGFR-2 inhibitors on resolving the development of sorafenib resistance in liver cancer via AKT overactivation.

## Figures and Tables

**Table 1:** MTT assay results as IC_50_ (μM) on HepG2, PC-3, and selectivity index on noncancerous cell line (WI38).

	Cytotoxicity in IC_50_ (μM) ^a,#^			
Compound	HepG2	PC-3	WI38	Selectivity Index (SI) ^b^
**3a**	7.96 ± 0.37	29.59 ± 1.66		
**3b**	3.11 ± 0.14	2.15 ± 0.12	12.46 ± 0.68	5.79
**3c**	107.00 ± 4.94	10.53 ± 0.59		
**3d**	13.41 ± 0.62	19.02 ± 1.06		
**3e**	22.38 ± 1.03	39.69 ± 2.22		
**3f**	4.30 ± 0.20	7.47 ± 0.42		
**3g**	3.77 ± 0.17	20.53 ± 1.15		
**4a**	41.07 ± 1.90	1.84 ± 0.10		
**4b**	34.19 ± 1.58	19.32 ± 1.08		
**4c**	3.02 ± 0.14	3.12 ± 0.17	35.33 ± 1.92	11.68
**6a**	9.80 ± 0.45	0.50 ± 0.03		
**7a**	27.32 ± 1.26	81.06 ± 4.54		
**7b**	2.48 ± 0.11	26.61 ± 1.49		
**7c**	39.60 ± 1.83	9.72 ± 0.54		
**7d**	8.91 ± 0.41	6.35 ± 0.36		
**Doxorubicin**	2.09 ± 0.10	2.53 ± 0.14	9.84 ± 0.54	4.69

^a^ IC_50_ is the mean ± S.D. (*n* = 3). ^#^ *p* < 0.005. ^b^ SI is calculated by dividing the IC_50_ of WI38/PC-3. SI > 2 is considered safe [44].

**Table 2:** The effect of **3b** and **4c** treatment on cell percentage in different cell cycle phases.

		% Content of DNA			
Compounds	G0:G1	S	G2:M	PreG1	Comment
**3b**/HepG2	39.42	56.19	4.39	42.06	cell growth arrest@ S
**4c**/HepG2	53.28	45.24	1.48	29.81	cell growth arrest@ G1/S
**cont.** HepG2	49.72	41.29	8.99	1.74	-

**Table 3:** Apoptotic cells percentage after treating HepG2 with **3b** and **4c**.

Compounds	Apoptosis			Necrosis
	Total	Early	Late	
**3b**/HepG2	42.06	2.51	24.53	15.02
**4c**/HepG2	29.81	3.85	16.55	9.41
**cont.** HepG2	1.74	0.55	0.17	1.02

## Data Availability

Data are contained within the article.
